# Comparative effectiveness of home blood pressure telemonitoring (HBPTM) plus nurse case management versus HBPTM alone among Black and Hispanic stroke survivors: study protocol for a randomized controlled trial

**DOI:** 10.1186/s13063-015-0605-5

**Published:** 2015-03-15

**Authors:** Tanya M Spruill, Olajide Williams, Jeanne A Teresi, Susan Lehrer, Liliana Pezzin, Salina P Waddy, Ronald M Lazar, Stephen K Williams, Girardin Jean-Louis, Joseph Ravenell, Sunil Penesetti, Albert Favate, Judith Flores, Katherine A Henry, Anne Kleiman, Steven R Levine, Richard Sinert, Teresa Y Smith, Michelle Stern, Helen Valsamis, Gbenga Ogedegbe

**Affiliations:** New York University School of Medicine, 227 East 30th Street, Room 640, 10016 New York, NY USA; Department of Neurology, Columbia University Medical School, New York, NY USA; Research Division, Hebrew Home at Riverdale, Bronx, NY USA; Columbia University Stroud Center and New York State Psychiatric Institute, New York, NY USA; Health and Hospitals Corporation, New York, NY USA; Department of Medicine, Medical College of Wisconsin, Milwaukee, WI USA; Office of Clinical Research, National Institutes of Health, National Institute of Neurological Disorders and Stroke, Bethesda, MD USA; Department of Neurology, New York University School of Medicine, New York, NY USA; Woodhull Medical Center, New York, NY USA; Bellevue Hospital Center, New York, NY USA; Harlem Hospital, New York, NY USA; Department of Neurology, SUNY Downstate Medical Center, Brooklyn, NY USA; Department of Emergency Medicine, SUNY Downstate Medical Center, Brooklyn, NY USA; The Department of Neurology, Kings County Hospital Center, Brooklyn, NY USA; Department of Emergency Medicine, Kings County Health Center, Brooklyn, NY USA; Jacobi Medical Center, New York, NY USA; Neurology Service, Kings County Hospital, Brooklyn, NY USA

**Keywords:** stroke, hypertension, blood pressure, disparities, telehealth, comparative effectiveness research

## Abstract

**Background:**

Black and Hispanic stroke survivors experience higher rates of recurrent stroke than whites. This disparity is partly explained by disproportionately higher rates of uncontrolled hypertension in these populations. Home blood pressure telemonitoring (HBPTM) and nurse case management (NCM) have proven efficacy in addressing the multilevel barriers to blood pressure (BP) control and reducing BP. However, the effectiveness of these interventions has not been evaluated in stroke patients. This study is designed to evaluate the comparative effectiveness, cost-effectiveness and sustainability of these two telehealth interventions in reducing BP and recurrent stroke among high-risk Black and Hispanic stroke survivors with uncontrolled hypertension.

**Methods/Design:**

A total of 450 Black and Hispanic patients with recent nondisabling stroke and uncontrolled hypertension are randomly assigned to one of two 12-month interventions: 1) HBPTM with wireless feedback to primary care providers or 2) HBPTM plus individualized, culturally-tailored, telephone-based NCM. Patients are recruited from stroke centers and primary care practices within the Health and Hospital Corporations (HHC) Network in New York City. Study visits occur at baseline, 6, 12 and 24 months. The primary outcomes are within-patient change in systolic BP at 12 months, and the rate of stroke recurrence at 24 months. The secondary outcome is the comparative cost-effectiveness of the interventions at 12 and 24 months; and exploratory outcomes include changes in stroke risk factors, health behaviors and treatment intensification. Recruitment for the stroke telemonitoring hypertension trial is currently ongoing.

**Discussion:**

The combination of two established and effective interventions along with the utilization of health information technology supports the sustainability of the HBPTM + NCM intervention and feasibility of its widespread implementation. Results of this trial will provide strong empirical evidence to inform clinical guidelines for management of stroke in minority stroke survivors with uncontrolled hypertension. If effective among Black and Hispanic stroke survivors, these interventions have the potential to substantially mitigate racial and ethnic disparities in stroke recurrence.

**Trial registration:**

ClinicalTrials.gov NCT02011685. Registered 10 December 2013.

## Background

Despite progress in the reduction of stroke mortality for the general U.S. population [1], Blacks and Hispanics continue to experience worse stroke-related outcomes compared with whites, including higher rates of recurrent stroke [2-8]. While the reasons for this disparity are complex, epidemiologic studies show that Black and Hispanic stroke survivors have worse secondary stroke risk factor profiles compared with whites, including higher rates of uncontrolled hypertension, diabetes, and hyperlipidemia [9-14]. Of these risk factors, hypertension is the most important intervention target [15]; a systematic review found that a 10-mm Hg reduction in systolic blood pressure (SBP) was associated with a 31% reduction in risk of recurrent stroke [16]. There is evidence that the impact of elevated SBP on stroke risk is three times greater for Blacks than for whites [17]. Thus, interventions targeting BP reduction in minorities have the potential to substantially mitigate racial and ethnic disparities in stroke recurrence.

Both medical and behavioral interventions have been proven effective in improving BP control in hypertensive patients [18]; however, translation of this evidence to improved health outcomes is suboptimal. Among stroke patients, poor BP control results from barriers that exist at multiple levels of care including the patient, the physician, and the healthcare system [19-21]. Up to one-third of stroke patients report poor adherence to antihypertensive medications, which is associated with increased risk of death after stroke [22] and is more common in minority patients compared with white patients [23,24]. Poor adherence to hypertension treatment guidelines is another significant barrier, particularly in the care of Black patients [25,26]. Even when providers adhere to guidelines, physicians often lack appropriate aggressiveness in the use of antihypertensive medications [27-29]. With respect to the healthcare system, Black and Hispanic patients with stroke report poorer access to care and medications compared with their white counterparts [30,31]. Further, poor integration of clinical decision support tools into daily practice and lack of integration of self-management interventions with robust health information technology platforms may contribute to suboptimal BP control in minority populations [32,33]. Improving BP control and reducing recurrent stroke among minority stroke survivors will require complex strategies that address barriers at each of these levels.

Two such interventions are (1) home BP telemonitoring (HBPTM) that provides regular feedback on BP levels to patients and their providers, which engages patients in their care, improves medication adherence, and reduces clinical uncertainty and inertia [34-36]; and (2) telephonic nurse case management (NCM), which provides education and individual support to enhance patient adherence to prescribed medications, self-management behaviors, and patient-provider collaboration [37-40]. Clinical trial evidence of the effectiveness of HBPTM and NCM for BP reduction is well documented. However, their widespread implementation in primary care practices has been hindered by the lack of scalability and sustainability, their labor-intensive nature, the lack of integration into the primary care infrastructure, and the absence of data on their comparative and cost-effectiveness. Further, the utility of these interventions among Black and Hispanic stroke patients remains unproven. The proposed practice-based stroke telemonitoring hypertension trial is designed to address these critical gaps in the literature.

### Study aims

The aim of this study is to evaluate the comparative effectiveness, cost-effectiveness and sustainability of home BP telemonitoring (HBPTM) alone versus HBPTM plus tailored, telephone-based NCM to reduce BP and prevent recurrent stroke among Black and Hispanic stroke survivors with uncontrolled hypertension. The combined HBPTM + NCM intervention we are evaluating is the ‘House Calls’ program offered through the New York City Heath and Hospitals Corporation (HHC). This established telehealth program utilizes an interactive web-based system to integrate HBPTM with personalized care, education and disease management from experienced nurses and has been shown to achieve significant reductions in BP [41]. There is evidence suggesting that the combination of home BP monitoring and NCM may be more effective in reducing BP than either intervention alone, particularly with respect to sustainability of effects [42,43].

## Methods/Design

### Overview of study design

This is a practice-based, multisite comparative and cost-effectiveness randomized controlled trial. Each patient will be randomly assigned to one of two 12-month interventions: 1) home BP telemonitoring (HBPTM) or 2) HBPTM plus tailored telephone-based nurse case management (HBPTM + NCM). We will recruit 450 Black and Hispanic patients with nonsevere poststroke disability and uncontrolled hypertension from the HHC Network - a municipal healthcare system that serves more than 1.8 million urban and diverse patients.

**Figure 1:**
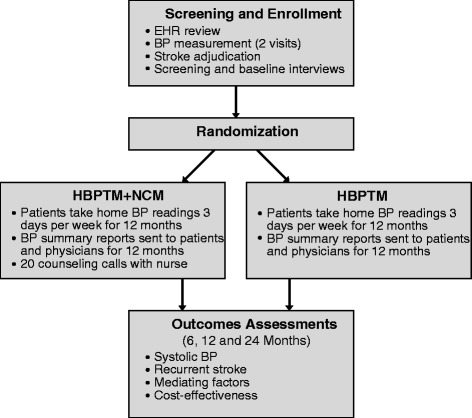
**Study design.**

The primary hypothesis is that supplementing HBTPM with tailored NCM will lead to greater reduction in SBP and stroke recurrence rate than HBPTM alone at 12 and 24 months, respectively. The secondary hypothesis is that the combined HBPTM + NCM intervention will be more cost-effective than HBPTM alone in reducing SBP and recurrent stroke. In exploratory analyses, we will assess changes in physicians’ treatment decisions, patients’ health behaviors, and other stroke risk factors, and will evaluate their role as mediators of intervention effects. The study design is shown in Figure 1.

### Research setting

The New York City Health and Hospitals Corporation (HHC) is an integrated municipal healthcare delivery system that serves more than 1.4 million New Yorkers every year, of whom more than 475,000 are uninsured. It is the largest municipal healthcare organization in the United States. Many HHC facilities are designated AHA Stroke Centers of Excellence, where stroke patients receive quality care and urgent diagnosis and treatment. The stroke telemonitoring hypertension trial is being implemented at the following HHC hospitals: Jacobi Medical Center, Bellevue Hospital Center, Harlem Hospital Center, Kings County Hospital Center and Woodhull Medical Center. The study protocol has been approved by the Institutional Review Boards of New York University School of Medicine (i13-00281), the Biomedical Research Alliance of New York (BRANY; 13-02-227 (HHC)-11), the Medical College of Wisconsin (PRO00019839) and the Hebrew Home for the Aged at Riverdale (0312I/P084/02).

### Patient population

Eligibility criteria were chosen to be as inclusive as possible while accounting for patients’ ability to participate in the interventions and to complete the 2-year study. The following inclusion and exclusion criteria are evaluated during a multistep screening process using a combination of EHR review, patient self-report and external stroke adjudication.

#### Inclusion criteria

Inclusion criteria are as follows:

- Age 18 years or older
- Black or Hispanic
- Fluent in English or Spanish
- At least one month post-discharge for ischemic or hemorrhagic stroke
- Modified Rankin Scale [44] score of ≤3, indicating nonsevere poststroke disability
- Screening SBP ≥140 mm Hg, defined by the average of three BP readings taken at each of two visits
- Patient plans to continue receiving care at the study site for the next two years

#### Exclusion criteria

Exclusion criteria are any of the following:

- Moderate to severe cognitive impairment as indicated by a score of ≥6 on the Comprehensive Assessment and Referral Evaluation (CARE)-Diagnostic Scale [45-47]
- Significant psychiatric comorbidity (indicated in EHR or self-reported)
- Significant verbal speech impairment (unable to complete screening)
- Unable to comply with the HBPTM and/or NCM protocols (either self-selected or apparent during screening that patient could not complete all required tasks)
- Upper arm circumference ≥52 cm, the maximum limit of the extra-large BP cuff
- Dialysis or diagnosis of end stage renal disease (indicated in EHR or self-reported)
- Relocating out of area or extended travel during study period
- Participation in other interventional clinical trials
- Pregnancy

### Study procedures

#### Recruitment

Patients are recruited using two main approaches: (1) identification of potentially eligible patients from Stroke Center admission and discharge lists and (2) ongoing review of the electronic health records (EHRs) at HHC primary care or outpatient neurology/stroke practices using ICD-9 codes for hypertension and stroke. At the beginning of the study at each study site, the project coordinator works with clinic leadership to develop a process for recruitment at that site. Clinic staff members are given a laminated pocket card with the eligibility criteria and research assistant (RA) contact information to facilitate referrals. Physicians of potentially eligible patients are asked to describe the study to their patients briefly and obtain permission for research staff to contact the patients. Those who are interested and potentially eligible are scheduled for screening.

#### Screening and baseline visits

All interactions between the RA and the patient are in English or Spanish, depending on the patient’s preference. After obtaining written informed consent, the RA takes three BP measurements using the Microlife Watch BP Office, a validated automated BP device (see www.dableducational.org), following American Heart Association guidelines [48]. Patients with an average SBP ≥140 mm Hg are scheduled for a second eligibility visit within 3 to 14 days, at which time a second BP measurement is taken. Patients must have an average SBP ≥140 mm Hg at each of the two screening visits to be eligible. To assess other eligibility criteria, the RA administers a computer-assisted personal interview (CAPI) programmed by the Research Core (see Data Management section below), which includes the CARE-Diagnostic Scale, modified Rankin Scale and other self-report items. Patients who are eligible are scheduled for a baseline visit, at which time the RA takes measurements of height, weight, waist circumference and BP and administers the baseline interview; self-report measures included in this interview are listed in the Study Measures section below.

#### Randomization

Once baseline assessments are completed, all relevant records related to the patient’s stroke admission are abstracted from the EHR and sent to the adjudication committee for review. Once the adjudicators have confirmed the diagnosis of stroke, the Research Core performs the randomization procedure. The RA informs the patient of the assigned study arm and enters him/her into the telehealth web portal, which triggers delivery of the HBPTM device to the patient’s home and contact from a NCM. Randomization is within primary care physician on a 1:1 assignment to the two treatment arms.

#### Follow-up visits

The RA meets with the patient at 6, 12 and 24 months to collect self-report data and BP measurements using the automated BP device. Patients’ EHRs are also reviewed at each time point. Visits are scheduled to coincide with regularly scheduled appointments at the patient’s hospital, but may be conducted in the patient’s home if needed.

#### Compensation

Patients receive $10 compensation for each of the two eligibility visits, $30 for the baseline visit, $40 for the 6-month visit, $50 for the 12-month visit and $50 for the 24-month visit, for a possible total compensation of $190.

#### Data and safety monitoring plan

Adverse events are reported to the appropriate institutional review boards (IRBs) and Privacy Boards. To ensure the safety of participants and the validity and integrity of the data, a data and safety monitoring board (DSMB) was established and meets approximately every six months. The DSMB includes senior investigators with expertise in stroke, hypertension, and biostatistics.

### Study interventions

#### Home blood pressure telemonitoring

Participants in both groups receive an automated home BP monitoring device (A&D UA-767 Plus) that has been validated for the measurement of BP by the British Hypertension Society and is recommended at www.dableducational.org. This device has the telemonitoring capability to transfer patients’ BP measurements wirelessly to a secure server in real time, without any action by the patient. The BP readings can then be disseminated to intended targets: the research team, the NCMs, and patients’ physicians. The patient also sees the BP readings when she or he takes the measurements, and is asked to keep a paper-and-pencil log of the readings, which has been shown to enhance self-management and adherence. For safety measures, the monitors are pre-programmed with BP alarm values (below 90 and/or 55 mmHg or above 180 and/or 110 mmHg), which when triggered, activate an e-mail or SMS message to the research staff or NCM, depending on study group assignment, prompting follow-up with the patient. All patients are trained by an RA in the use of the HBPTM device and instructed to take their BP twice in the morning and twice in the evening, three days a week during the 12-month intervention.

Patients randomized to the HBPTM alone group receive printed educational materials on management of hypertension and stroke developed by NHLBI in English or Spanish as preferred. The NCM contacts patients within 2 weeks to make sure they are comfortable using the HBPTM device and to review the educational materials. The patient’s physician receives home BP reports via secure email before every scheduled appointment for the duration of the study. At the end of the study, the patient will be asked to return the home BP monitor to study staff. All BP readings stored in the actual monitor will be deleted.

#### Home blood pressure telemonitoring plus nurse case management

The combined intervention, the HouseCalls telehealth program, is integrated into the HHC system as part of its home care program. The intervention is delivered by HHC nurses who have real-time access to patients’ EHRs and are in communication with their providers. After randomization and delivery of the HBPTM device to patients’ homes, the NCM contacts patients within two weeks to make sure they are comfortable using the device. The NCM then initiates the planned schedule of counseling phone calls: weekly calls for months 1 to 2, biweekly calls for months 3 to 4, and then monthly calls for months 5 to 12; more frequent calls may be completed at the NCMs discretion. Each call lasts between 15 and 45 minutes depending on the needs of the patient. The NCM has access to patients’ home BP data via a secure website, where the BP readings are displayed in easy-to-read charts and figures that highlight the control rate for each week. This information is used by the NCM as a basis for the counseling sessions. Patients’ physicians have access to this website, and also receive home BP reports via secure email before every scheduled appointment for the duration of the study.

During the scheduled telephone sessions, the NCM provides self-management education and medication and appointment reminders, and facilitates patient-provider communication. The NCM works with the patient to set behavioral goals based on individual needs. Target behaviors discussed may include dietary changes, physical activity, weight loss, medication adherence, and smoking cessation. The NCM assesses the patient’s barriers to behavior change and use problem-solving and motivational interviewing techniques to support behavior change efforts. At the end of each counseling session, the NCM records the notes of the encounter in the web portal and communicates with the patient’s physician if needed (for example, regarding medication side effects or other problems reported by patients, or the need for appointments or medication refills). The NCM also reviews the patient’s clinical information and provides feedback of abnormal lab results. Thus, the intervention addresses patient-level issues (adherence, self-management), provider-level issues (clinical inertia), and system issues (web-based HBPTM integration into EHR, embedded NCM).

### Treatment fidelity

Our efforts to maximize the quality and consistency of the interventions include (1) careful training of patients in the use of the home BP monitors; (2) strategies to enhance patient adherence to each of the interventions (that is, reminder calls, monitoring transmission of home BP readings, weekend availability for NCM sessions); and (3) monitoring of adherence to the interventions to evaluate consistency across study sites and, for the HBPTM + NCM arm, across nurses. A key strength of this study is the use of an established, successful NCM intervention that does not require extensive training or supervision from research staff, aside from details of the study protocol. The nurses working with the House Calls program have undergone extensive training in principles and strategies of behavior change needed for effective counseling of chronic disease patients (for example, motivational interviewing skills, problem-solving approaches). To provide data on intervention content for secondary analyses, the nurses complete a checklist following each counseling session to indicate the topics covered.

### Data collection and management

This study has a Research Core that is responsible for the following activities: 1) development of the computer-assisted data collection system, 2) staff training and certification in data collection, 3) randomization procedures, 4) data monitoring and quality control, 5) data processing, and 6) data analysis. The Research Core prepares regular reports for internal and external monitoring of progress toward study milestones, and will provide blinded and unblended data requests for DSMB meetings.

### Study measures

The primary outcomes are within-patient change in SBP from baseline to 12 months and stroke recurrence rates at 24 months. Secondary outcomes are 12- and 24-month cost-effectiveness. Exploratory outcomes include 1) physicians’ hypertension medication intensification [49]; (2) patients’ health behaviors (diet, physical activity, medication adherence); and (3) other important stroke risk factors (blood glucose, lipids).

#### Blood pressure

BP is measured at each study visit by a trained RA using a validated automated BP monitor (Microlife WatchBP Office), following the American Heart Association (AHA) guidelines [48]. The device is programmed to take three readings at 2-minute intervals after an initial rest period of 3 minutes. The patient is asked to sit quietly without talking during the measurement period. The three automated readings are averaged to give the final SBP measurement for each visit.

#### Stroke recurrence

All participants are urged to contact research staff as soon as any event that might be a stroke has occurred. If research staff members learn from a participant, family member, NCM or physician that a stroke might have taken place, all clinical, laboratory and imaging data needed to confirm the event are extracted from the EHR by the RA and sent to the Research Core within one week. These materials are then provided to the two blinded adjudicators for independent review using the National Institute of Neurological Disorders and Stroke (NINDS) Stroke Adjudication Worksheet [50,51]. Final reports regarding the event will be returned to the Research Core within 15 days. If the two adjudicators have different opinions, the materials will be sent to a third adjudicator. The Research Core records the final decision.

#### Self-report measures

Patient interviews at baseline, 6 months, 12 months and 24-months are the source of data for analyses of other study outcomes. The selection of study measures was guided by the NINDS Stroke Common Data Elements (CDEs) [51] in order to promote standardization with the larger body of stroke research. Some of the measures we require to test our hypotheses are not included in the Stroke CDEs; for these we selected widely used, validated measures. All study materials not already available in Spanish were translated by a professional translation service. The interview instrument includes measures of 1) demographics and socioeconomic status; 2) behavioral history (alcohol, nicotine, drug use); 3) family medical history; 4) Charlson Comorbidity Index [52]; 5) executive function (Frontal Assessment Battery) [53]; 6) depression (PROMIS Depression short-form) [54]; 7) disability (Modified Rankin Scale [44]); 8) functional status (the Barthel Index [55]); 9) health-related quality of life (EuroQoL [56]); 10) medication adherence (Morisky Medication Adherence Scale [57]); 11) diet (NCI Percentage Energy from Fat Screener) [58]; 12) physical activity (International Physical Activity Questionnaire) [59]; and 13) a healthcare utilization questionnaire that collects information on hospitalizations, emergency department (ED) visits, health insurance and medications developed by the investigators.

#### Anthropometric measurements

A stadiometer is used to measure height, a validated digital scale for weight, and a tape measure specifically designed for accurate and repeatable measurements of various body dimensions for waist circumference. All height and waist circumference measurements are recorded to the nearest 0.1 cm and all weight measurements are recorded to the nearest 0.1 kg respectively. All measurements are taken without shoes and with light clothes.

#### Electronic health record data

Patients’ electronic health records (EHRs) are reviewed following each study visit to extract information regarding hypertension characteristics and treatment (that is, medications, dosages, and physician notes), comorbidity, and relevant lab results (that is, blood glucose, lipids). EHRs are also the source of information for certain cost data, including direct costs to third party payers.

#### Cost data

In addition to expenses associated with implementation of the two interventions, cost measures include resource costs associated with direct health care costs, including use of ambulatory care services, such as physician and ED visits, laboratory tests, hospitalizations, surgical procedures, and medication regimens. Intervention costs will include all direct (labor) and indirect (overhead) incremental costs associated with implementation of the interventions - most notably, labor and equipment costs associated with HBPTM, costs of producing and distributing educational materials, and costs associated with the NCM’s time. Unit costs of interventions will be measured by detailed resource-cost analyses of the time of involved health professionals and supplies or equipment. Utilization of health care services is obtained from EHRs and patient surveys. The unit costs of ambulatory services are obtained from clinic billing records and the costs of medications from prescribed regimens and the Red Book prices for the prevailing years.

### Sample size and power analysis

The primary hypotheses are (a) a greater reduction in systolic BP over three waves of data: baseline, 6 and 12 months and (b) a lower 24-month stroke recurrence rate among the combined HBPTM + NCM group compared to the HBPTM alone group. Although there may be more than one episode of stroke per patient, given the sample size, we will combine all events. We assume separate analyses of the outcomes, with pre-specified α = 0.05 for each outcome. Statistical modeling procedures will be used that allow the inclusion in the analysis of participants who do not complete the follow-up assessment (on an intent-to-treat basis). The approach to the power calculations incorporates the design feature of clustering. The cluster size of participants who are eligible (uncontrolled hypertension with a history of stroke) within PCP is estimated to range from 5 to 6. Data from a similar study of telemedicine (IDEATel) delivered to New York City Hispanics and Blacks did not show heterogeneous variances [60], so this scenario was not included in power calculations. Specific types of selection bias and attrition were considered.

Based on studies of telemedicine in Blacks and Hispanics [42,43,60-62], it was estimated that the baseline and longitudinal standard deviations of SBP at baseline will be between 18 and 22 mm Hg. The observed mean differences were from 3 to 8 for SBP. We posited that the enhanced telemedicine intervention will result in an effect size ranging from a 6- to 8-point difference in SBP. The assumptions were as follows: α = .05 for a two-tailed test; 1-β = .80 and above; and δ = μ_1_-μ_2_ = 6, 7, 8 (SBP point reduction). The following assumptions were used in the power calculations: R = 0.90 (reliability); g = 2 (groups), pooled σ = 20.75 (baseline control: 19.6; intervention 19.2; 6 month control: 21.7; intervention: 20.5; 12 month control: 21.0; intervention: 22.3), d = δ/σ (where δ is difference in end study and d is Cohen’s d;) [63]. The cluster size was estimated as 5; the intracluster correlation (ICC) = 0.03; and Vif = 1+ (clustersize-1)*ICC = 1.12.

### Power for comparing rates of change in systolic blood pressure response over 12 months between groups

The power to detect a difference in slopes (β_1A_-β_1B_) over the twelve months of the study (using all waves of data) was examined. An estimate of the required sample sizes [64,65], is given, considering time measured as the duration between the first and jth occasion, j = 0, 0.5, 1 year. The assumptions above were used regarding design effects and reliability; these calculations assume that ρ = 0.5, 0.6 (the average correlation between baseline and follow up) and δ = 6, 7 (SBP = yearly point reduction). Based on examination of endpoint differences, the required sample sizes ranged from 175 to 225, depending on the assumptions regarding effect sizes [66]. The selected sample size of 225 per group permits detection of relatively small effect sizes, ranging from 5 to 6, depending on different scenarios.

#### Power for the stroke recurrence outcome at 24 months

Power was examined for stroke recurrence outcomes using the sample size formula for the log-rank test, where *d* is the expected probability of an event over subjects and is the hazard ratio. An additional calculation examining time-to-event using a Cox model was also performed. The assumptions were as above regarding reliability, cluster size and intracluster correlation coefficient. The correlation between pre- and post-measures was set at 0.60. A sample size of between 208 and 223 per group is needed, depending on the model and assumptions for 80% power to detect a 10% intervention group reduction in the rate of stroke at 24 months from a baseline rate of 15% [67].

#### Summary

Conservatively, under the assumptions specified above, 225 subjects per group will provide power ≥0.80 to detect the hypothesized 6 to 8 unit differential change in SBP, based on testing the Time X Group interaction in a mixed model, adjusting for unreliability, design effects due to clustering and serial correlations. This sample size will also provide power ≥ 0.80 to detect the hypothesized 10% group reduction in the rate of stroke at 24 months from a baseline rate of 15%.

### Analytic plan

#### Primary hypotheses: patients randomized to HBPTM + NCM will have (a) greater reduction in SBP at 12 months, and (b) lower rates of stroke recurrence at 24 months, compared to those in HBPTM alone

The analyses of SBP change will use mixed random effects models, and a full information maximum likelihood approach, with sensitivity analyses using generalized estimating equations. The change from pre- to post-treatment values of continuous outcomes will be modeled as functions of treatment group, time and the interaction of time and treatment. The intent-to-treat analyses performed using SAS PROC MIXED will permit modeling design effects (clustering), and allow for the possible group heterogeneity in residual variances and serial correlations that may require modeling to satisfy model assumptions and improve model fit. Based on prior analytic experience with the outcome variables, it is not expected that transformations will be necessary. Analyses of 24-month stroke recurrence will be performed using the log rank test or a Cox model examining time-to-event, should covariates be required.

Prior to analyses, baseline values of all variables from each arm will be examined; however, no *P* values will be provided, and covariates are not proposed for inclusion in the main analyses of treatment effects. If one or more sources of potential (selection or attrition) bias are identified, the predicted values from those analyses will be included as covariates in secondary analyses. Depending on the severity of missing data, other modeling techniques may be used.

Since the analysis and inference is based on intent-to-treat, an attempt will be made to obtain post-treatment data from all randomized participants, regardless of their adherence to the interventions. Nonetheless, secondary analyses will be conducted to investigate the impact of differential participation, stratifying participants in the treatment conditions based on their degree of participation and examining differences between strata on the outcome measures at follow-up.

#### Secondary hypotheses: HBPTM + NCM will be more costly but also more cost-effective in reducing (a) SBP at 12 months and (b) stroke recurrence at 24 months than HBPTM alone

The principal measures of cost to be used in the study pertain to (i) intervention-related resource costs and (ii) overall direct health care costs. To assess cost differences in intervention and overall costs across interventions, we will first compare average patient costs across the two groups. Patient-level analyses of costs, similar to those described above for outcomes, will be used to produce regression-adjusted measures of cost-effectiveness for each intervention. Using methods consistent with the standards published in 1996 [68-70], we will conduct incremental cost-effectiveness analyses (CEAs) to assess the relative effects of the interventions on three time periods: intervention period (12 months), follow-up period (24-months post-randomization), and lifetime perspective. Measures of effectiveness in the intervention and follow-up period CEAs will be based on differences across intervention groups in (1) mean changes in SBP (mm Hg); (2) stroke recurrence; and (3) quality of life (as measured by EuroQoL scores). Bootstrapping procedures will be applied to obtain confidence intervals around all of our cost-effectiveness estimates. Sensitivity analyses will be used to examine key uncertainties about CEA findings by identifying variables that have the most dramatic effects on estimated CE ratios and examining the effects of changes in these measures/assumptions on CEA results.

#### Exploratory analysis of potential mediators and moderators

We will conduct exploratory analyses to test potential mediating pathways involving (1) changes in other stroke risk factors (that is, lipids, blood glucose); (2) changes in health behaviors (that is, diet, physical activity, medication adherence); and (3) antihypertensive medication intensification (that is, adding or changing dose or class of medications). We will first compare effects of the two interventions on each of these outcomes, following the analysis plans detailed above for continuous and dichotomous variables as appropriate. We will then use formal tests to examine the extent to which the intervention effects are mediated through these variables. Since we have no reason to assume that effects of the interventions will differ by race/ethnicity or by site, our primary analyses do not stratify by these factors or include interaction effects. That said, we will conduct exploratory analyses to examine whether or not the interventions are equally effective for Black and Hispanic patients, and across study sites [71-73].

## Discussion

Disparities in stroke remain a major public health problem in the United States. There is an urgent need for secondary prevention strategies that can help to reduce disproportionately higher rates of recurrent stroke among Black and Hispanic patients. Many interventions to improve BP control have been developed and tested, and several have been proven effective under controlled conditions [74-77]. However, if these interventions cannot find a place in primary care practices, their impact on public health will be negligible. Progress toward this goal has been hindered by lack of comparative effectiveness and cost-effectiveness data, particularly in minority populations, and insufficient utilization of health information technology that is needed to support their dissemination and long-term sustainability in community-based primary care practices where the majority of low-income minority patients receive their care. Two potentially viable interventions in this respect are home BP monitoring and telephone-based NCM, both of which have proven efficacy in improving BP control and may reduce the risk of recurrent stroke. Adoption of these interventions at the primary care level may also reduce the risk of incident stroke, which contributes even more heavily than recurrent stroke to disparities [78].

A major limitation of previous studies of NCM and home BP monitoring is their lack of sustainability and scalability. This is largely due to the intensive nature of the delivery of these interventions, which often requires multiple office visits and patient counseling sessions. The ongoing stroke telemonitoring hypertension trial addresses this limitation by using well-established web-based and wireless technologies, which are easily deployable, efficient and potentially cost-effective. The merging of two established and effective interventions along with the utilization of health information technology supports the sustainability of the HBPTM + NCM intervention and feasibility of its widespread implementation. Embedding care mangers within practices further facilitates sustainability particularly in the current climate of outcome-based payment reform. Cost-effectiveness analysis is an integral objective of this study given the enormous financial burden of stroke. The American Heart Association projects that direct costs of stroke will increase 238% from 2010 to 2030 and indirect costs will increase 73%; total costs are projected to increase from $53.9 billion in 2010 to $140 billion in 2030 [79]. Thus cost-effective secondary stroke prevention interventions have the potential to produce enormous savings, particularly in the care of minority patients who suffer disproportionately higher stroke-related morbidity and mortality.

If successful, these telehealth interventions can be relatively easily adapted and incorporated into the care of 1.4 million New Yorkers who receive care within the HHC network. MetroPlus, HHC’s 420,000-member health plan, already reimburses for the House Calls program among patients with poorly controlled diabetes and congestive heart failure. Demonstrating the comparative- and cost-effectiveness of this program among stroke patients with uncontrolled hypertension may facilitate the process of expanding coverage for this intervention for this high-risk population as well. Ultimately, we envision implementation beyond our study population across the larger HHC community and other local healthcare systems, as well as other urban communities that, like NYC, have large populations of underserved minorities who would benefit from secondary stroke prevention interventions.

## Trial status

Recruitment for this study began in December 2013 and is ongoing.

## Abbreviations

BP: blood pressure
CEA: cost-effectiveness analysis
EHR: electronic health record
HBPTM: home blood pressure telemonitoring
HHC: Health and Hospital Corporations
IRB: institutional review board
NCM: nurse case management
RA: research assistant
RCT: randomized controlled trial
SBP: systolic blood pressure
